# Impact of Environmental Conditions During IVF Laboratory Handling on Sperm Quality

**DOI:** 10.3390/ijms27177641

**Published:** 2026-08-26

**Authors:** Péter Czétány, András Balló, László Márk, Nelli Farkas, Árpád Szántó, Gábor Máté

**Affiliations:** 1 Urology Clinic, Clinical Centre, University of Pécs, 7621 Pécs, Hungary; ballo.andras@pte.hu (A.B.); szanto.arpad@pte.hu (Á.S.); mate.gabor@dunamentirek.hu (G.M.); 2 National Laboratory on Human Reproduction, University of Pécs, 7624 Pécs, Hungary; laszlo.mark@aok.pte.hu; 3 Tapolca Institute, Dunamenti REK Reproduction Center Ltd., 8300 Tapolca, Hungary; 4 Department of Analytical Biochemistry, Institute of Biochemistry and Medical Chemistry, Medical School, University of Pécs, 7624 Pécs, Hungary; 5 MTA-PTE Human Reproduction Scientific Research Group, 7624 Pécs, Hungary; 6 Institute of Bioanalysis, Medical School, University of Pécs, 7624 Pécs, Hungary; nelli.farkas@aok.pte.hu

**Keywords:** assisted reproductive technology, sperm handling, sperm quality, DNA fragmentation, oxido–reduction potential

## Abstract

Spermatozoa are highly susceptible to environmental stress because of their limited antioxidant capacity and DNA repair mechanisms. During assisted reproductive technology (ART), semen samples are exposed to various laboratory conditions; however, current guidelines provide limited recommendations regarding optimal environmental parameters for sperm handling. The aim of this study was to evaluate the effects of environmental factors encountered in the ART laboratory on classical sperm parameters (total motility, progressive motility, and vitality) and functional markers, including oxido–reduction potential (ORP) and DNA fragmentation (DF). Normozoospermic native semen samples (*n* = 8) were exposed for 1 h to different temperatures (4, 24, 37, and −80 °C), oxygen concentrations (21 and 5%), visible or UV light, and centrifugation (400× *g*). UV irradiation and exposure to −80 °C exerted the most detrimental effects on classical sperm parameters. While −80 °C did not significantly affect ORP or DF, UV irradiation caused the most pronounced increases in both parameters. 5% O_2_ and 4 °C preserved several sperm quality parameters more effectively than conventional laboratory conditions (21%, 24, 37 °C). These findings demonstrate that even short-term exposure to various laboratory conditions can substantially affect sperm quality. Modification of environmental conditions during sperm handling may therefore contribute to preserving sperm function during ART.

## 1. Introduction

Spermatozoa are highly specialized cells adapted exclusively for fertilization. During their maturation, they undergo significant structural reduction, resulting in the loss of several organelles such as the Golgi apparatus, endoplasmic reticulum, and ribosomes, as well as key cellular mechanisms including robust antioxidant defenses and DNA repair systems [1]. Consequently, mature sperm cells are particularly vulnerable to oxidative stress (OS). Their reduced cytoplasmic volume limits the presence of enzymatic antioxidants, rendering them less capable of neutralizing excessive oxidative damage. Under physiological conditions, spermatozoa generate low levels of reactive oxygen species (ROS), which are essential for normal processes such as signal transduction, DNA condensation, maturation, capacitation, hyperactivation, the acrosome reaction, and sperm–oocyte fusion. However, elevated ROS levels—originating from endogenous sources (e.g., mitochondrial dysfunction, immature spermatozoa, leukocytes, varicocele) or exogenous factors (e.g., smoking, alcohol consumption, obesity, infections, radiation (UV))—can have detrimental effects [2]. These include DNA fragmentation, lipid peroxidation, compromised membrane integrity, enzyme inactivation, and apoptosis, ultimately leading to impaired sperm parameters such as reduced concentration, motility, and normal morphology [3,4,5]. It is worth mentioning that the membrane of sperm is especially prone to peroxidation because of its high content of polyunsaturated fatty acids (PUFAs) [6]. PUFAs play an important role in maintaining membrane fluidity; however, their multiple carbon–carbon double bonds also make them particularly susceptible to oxidative attack by ROS. This process leads to the formation of toxic breakdown products, mainly highly reactive lipid aldehydes such as malondialdehyde (MDA) and 4-hydroxynonenal (4HNE) [7,8,9]. These molecules can compromise cell membrane integrity, resulting in impaired sperm motility and reduced membrane fusogenic capacity during fertilization [10]. 4HNE subsequently reacts with proteins and nucleic acids as well. Oxidation of cytoskeletal proteins, such as actin, tubulin and dynein, may impair cytoskeletal integrity, potentially leading to alterations in sperm morphology and impaired motility. 4-HNE can target enzymes involved in glycolysis, the citric acid cycle, oxidative phosphorylation, the electron transport chain, and β-oxidation. Furthermore, 4-HNE can promote electron leakage from mitochondria, thereby contributing to mitochondrial dysfunction and loss of mitochondrial membrane potential [8,11,12]. Collectively, these effects can disrupt sperm energy metabolism and mitochondrial function, ultimately contributing to impaired motility and potentially promoting activation of the intrinsic apoptotic pathway. Sperm chromatin reaches a high level of compaction through the replacement of histones of somatic cells by protamines during spermatogenesis [13,14]. Although this highly compacted chromatin protects sperm DNA from damage, mature spermatozoa have limited DNA repair capacity and undergo progressive transcriptional silencing during maturation, making them particularly vulnerable to oxidative stress. Following oxidative damage, ROS can induce oxidative base modifications in sperm DNA. Among the DNA repair mechanisms retained in mature spermatozoa, base excision repair, involving enzymes such as 8-oxoguanine DNA glycosylase 1 (OGG1), plays an important role in the recognition and removal of oxidized bases. OGG1 removes oxidized bases from DNA, generating an abasic site that cannot be fully repaired by the mature spermatozoon. Consequently, repair of the resulting DNA lesion is largely dependent on the oocyte after fertilization [15,16].

To counterbalance oxidative stress, both enzymatic and non-enzymatic antioxidant defense systems are present in sperm cells and seminal plasma. The primary enzymatic antioxidants include superoxide dismutase (SOD), catalase (CAT), and glutathione peroxidase (GPX), which are found intracellularly but are more abundant in seminal plasma. Non-enzymatic antioxidants present mainly in seminal plasma as well consist of naturally occurring compounds, including glutathione, vitamins C and E, carotenoids, coenzyme Q10, as well as trace elements such as zinc and selenium. These molecules primarily act as free radical scavengers, neutralizing ROS before they can interact with and damage essential biomolecules, including lipids, proteins, and DNA [10,17].

As highlighted above, redox homeostasis plays a critical role in maintaining sperm functional competence and has therefore emerged as a key factor in the pathophysiology of male infertility [2,18]. In recognition of this, Agarwal et al. (2019) proposed the term male oxidative stress infertility (MOSI) to describe a distinct clinical condition characterized by elevated oxidative stress levels accompanied by abnormal semen characteristics [19] (in the present study, abnormal semen parameters refer to values below the lower fifth percentile reference limits established by the WHO [20]). Despite the growing need for efficient ART in light of the global fertility crisis [21], their overall success rates remain relatively low (approximately 30–40%) [22]. Sperm quality is often an underestimated and debated factor in this context. Nevertheless, a substantial body of evidence suggests that sperm quality plays a pivotal role in ART outcomes. Moreover, semen samples routinely spend a substantial amount of time outside the controlled thermal and atmospheric environment of the male reproductive tract during in vitro fertilization (IVF) and related assisted reproduction procedures, being exposed to variable ambient temperature, room light, atmospheric oxygen tension, and mechanical stress (e.g., centrifugation) in the IVF laboratory. Most of the previous studies have examined individual environmental stressors in isolation (e.g., temperature alone or oxygen tension alone), often over longer exposure windows than are typical of routine laboratory handling, which limits their direct applicability to everyday IVF laboratory practice.

The aim of the present study was to systematically compare, within the same ejaculate samples, the short-term (1 h) effects of several environmental conditions relevant to laboratory handling, including routine laboratory conditions (24 °C, 37 °C, visible light, and 21% O_2_) and experimental stress conditions (4 °C, −80 °C, 5% O_2_, UV irradiation, and prolonged centrifugation at 400× *g* for 1 h), on classical sperm parameters (motility, progressive motility, and vitality) and functional markers (ORP and DF). We hypothesized that the tested environmental conditions would exert differential effects on sperm quality, with conditions involving extreme temperature, UV irradiation, or prolonged centrifugation expected to induce greater deterioration of sperm parameters and/or functional markers than routine laboratory conditions. This approach aimed to identify environmental conditions associated with deterioration, preservation, or improvement of sperm quality, thereby identifying those that may warrant prioritization for modification or avoidance in the IVF laboratory.

## 2. Results

### 2.1. Temperature

Except for the 4 °C condition, incubation of the samples at all other temperatures resulted in a significant decrease in total sperm motility (*p* < 0.001), with the most pronounced reduction observed at −80 °C. In contrast, storage at 4 °C led to a marked and statistically significant increase in total sperm motility (*p* < 0.05). However, when progressive motility was examined, even exposure to 4 °C resulted in a significant decrease (*p* < 0.001), similar to the other temperature conditions.

When comparing the effects of the different temperatures to the 24 °C condition, incubation at 37 °C caused a mild but significant decrease in total sperm motility (*p* < 0.05), whereas at −80 °C the same parameter dropped drastically (*p* < 0.001). Interestingly, incubation at 4 °C significantly enhanced total sperm motility compared with 24 °C (*p* < 0.001). Regarding progressive motility, a mild and a pronounced reduction were observed at 37 °C and −80 °C, respectively (*p* < 0.05 and *p* < 0.001), while incubation at 4 °C maintained progressive motility at a higher level than that measured at 24 °C (*p* < 0.01).

**Figure 1:**
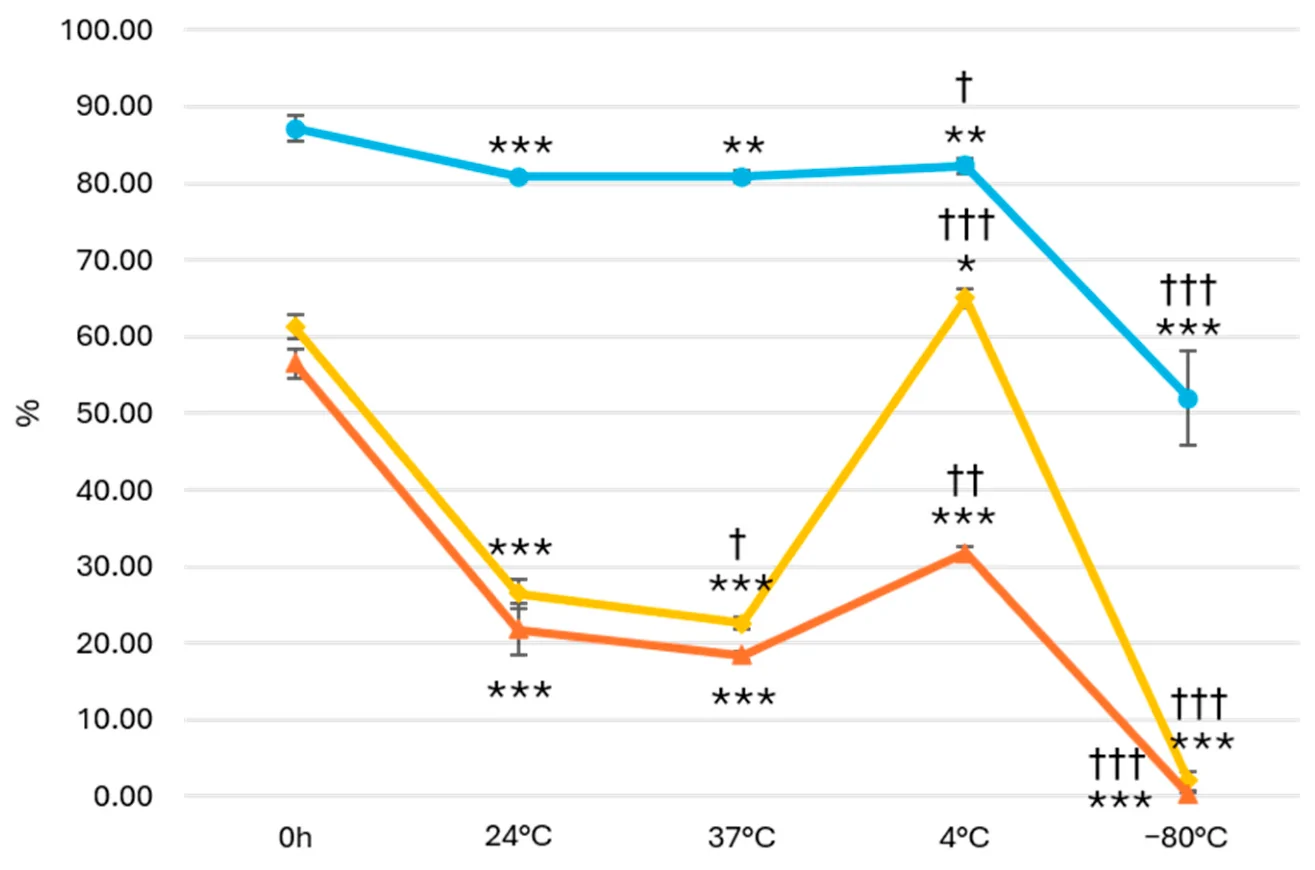
Effect of incubation temperature (24, 37, 4, −80 °C) on classical sperm parameters (motility (%) labeled with yellow, progressive motility (%) with orange, vitality (%) with blue; “asterisk” vs. 0 h, “dagger” vs. 24 °C) (Friedman-test, Bonferroni-Holm post hoc test, single “asterisk/dagger”: *p* < 0.05, double “asterisk/dagger”: *p* < 0.01, triple “asterisk/dagger”: *p* < 0.001).

A significantly lower sperm vitality was observed after 1 h of incubation at all tested temperatures, with the most substantial decline occurring at −80 °C (*p* < 0.001). However, compared with the 24 °C condition, only incubation at 4 °C and −80 °C resulted in a significant change in vitality: 4 °C preserved it at a slightly higher level, while −80 °C caused a strong reduction (*p* < 0.05 and *p* < 0.001, respectively) (Figure 1).

Significant changes in ORP levels compared with the baseline sample (0 h) were detected only at 37 °C and 4 °C. At body temperature (37 °C), ORP levels increased, whereas incubation at 4 °C resulted in a significant decrease (*p* < 0.001). The same pattern was observed when these conditions were compared with the 24 °C group (*p* < 0.001).

**Figure 2:**
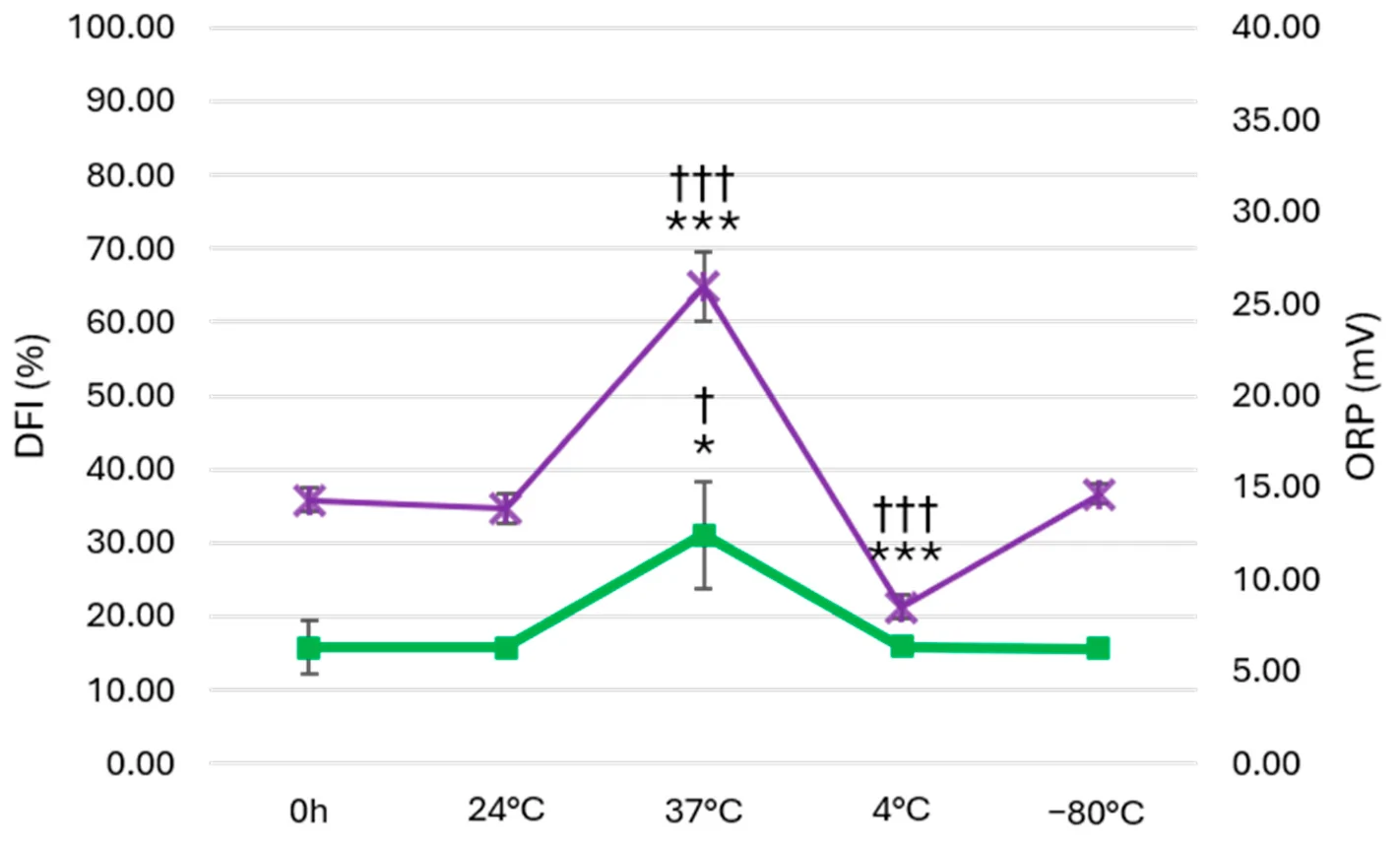
Effect of incubation temperature (24, 37, 4, −80 °C) on functional sperm parameters (ORP (mV) labeled with purple, DFI (%) with green; “asterisk” vs. 0 h, “dagger” vs. 24 °C) (Friedman-test, Bonferroni-Holm post hoc test, single “asterisk/dagger”: *p* < 0.05, triple “asterisk/dagger”: *p* < 0.001).

Regarding DF, only a mild but significant increase in the DNA fragmentation index (DFI) was detected at 37 °C compared with the control samples (0 h and 24 °C) (*p* < 0.05) (Figure 2).

### 2.2. Oxygen Concentration

In this experimental setting, samples incubated for 0 h and 37 °C served as control groups. Both atmospheric (21%) and reduced (5%) oxygen concentrations significantly decreased total and progressive motility compared with the 0 h control (*p* < 0.001). When compared with the 37 °C, total and progressive motility were preserved at significantly higher levels under 5% O_2_ exposure (*p* < 0.05 and *p* < 0.001, respectively). When comparing the effects of 21% and 5% oxygen, the 5% concentration resulted in a significantly higher rate of total motility (*p* < 0.001); however, no significant difference was observed in progressive motility.

**Figure 3:**
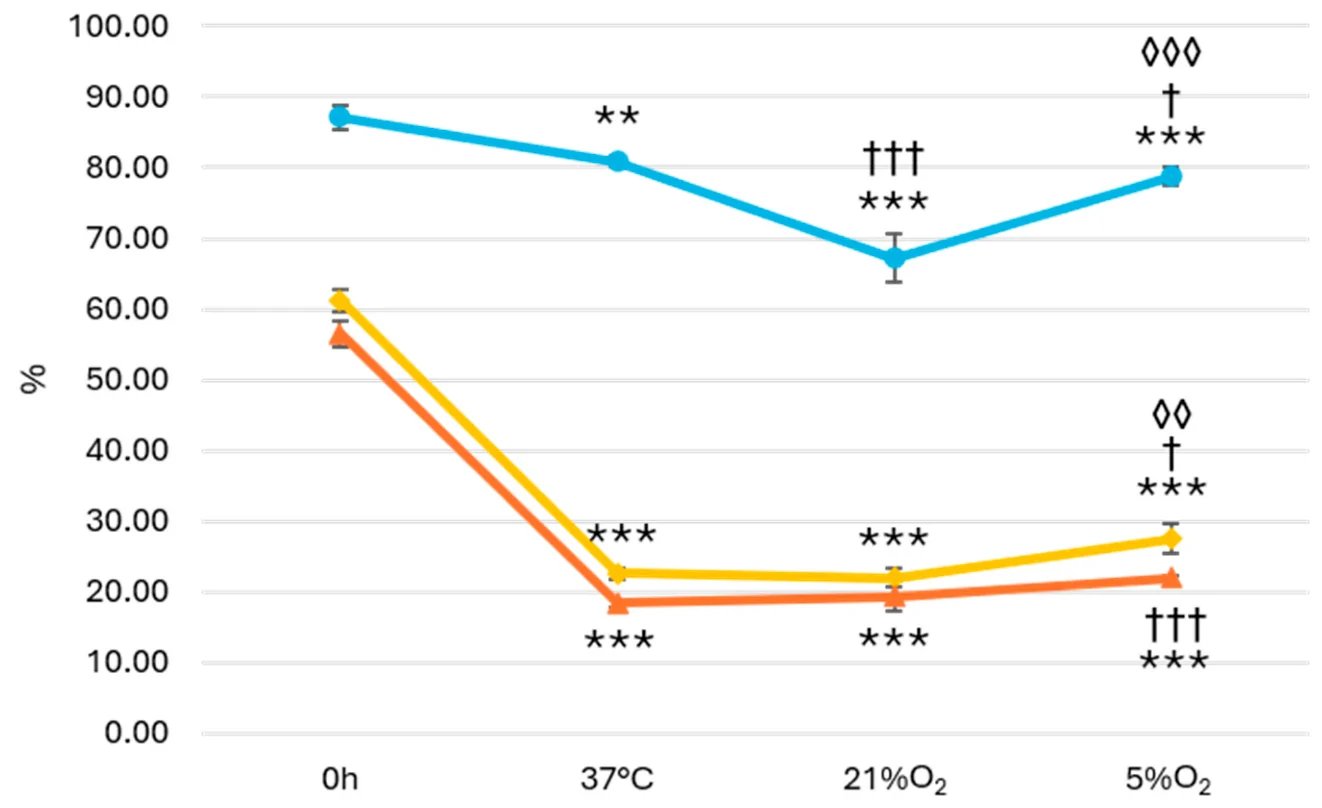
Effect of oxygen concentration (21 and 5%) on classical sperm parameters (motility (%) labeled with yellow, progressive motility (%) with orange, vitality (%) with blue; “asterisk” vs. 0 h, “dagger” vs. 37 °C, “diamond” vs. 21%O_2_ (Friedman-test, Bonferroni-Holm post hoc test, single “dagger”: *p* < 0.05, double “asterisk/diamond” *p* < 0.01, triple “asterisk/dagger/diamond” *p* < 0.001).

Exposure of the samples to both 21% and 5% oxygen concentrations significantly reduced vitality compared with the 0 h control (*p* < 0.001). When compared with the 37 °C control, 21% O_2_ induced a more pronounced reduction in vitality (*p* < 0.001) than 5% O_2_ (*p* < 0.05), although both effects were statistically significant. Direct comparison of the two gas concentrations revealed that vitality was better preserved at the lower (5%) oxygen concentration (*p* < 0.001) (Figure 3).

Incubation of samples at both oxygen concentrations for 1 h resulted in significantly elevated ORP values compared with the 0 h control (*p* < 0.001). However, only the 21% O_2_ condition generated significantly higher ORP values compared with the 37 °C control (*p* < 0.001). No statistically significant difference was observed between the 21% and 5% oxygen conditions.

**Figure 4:**
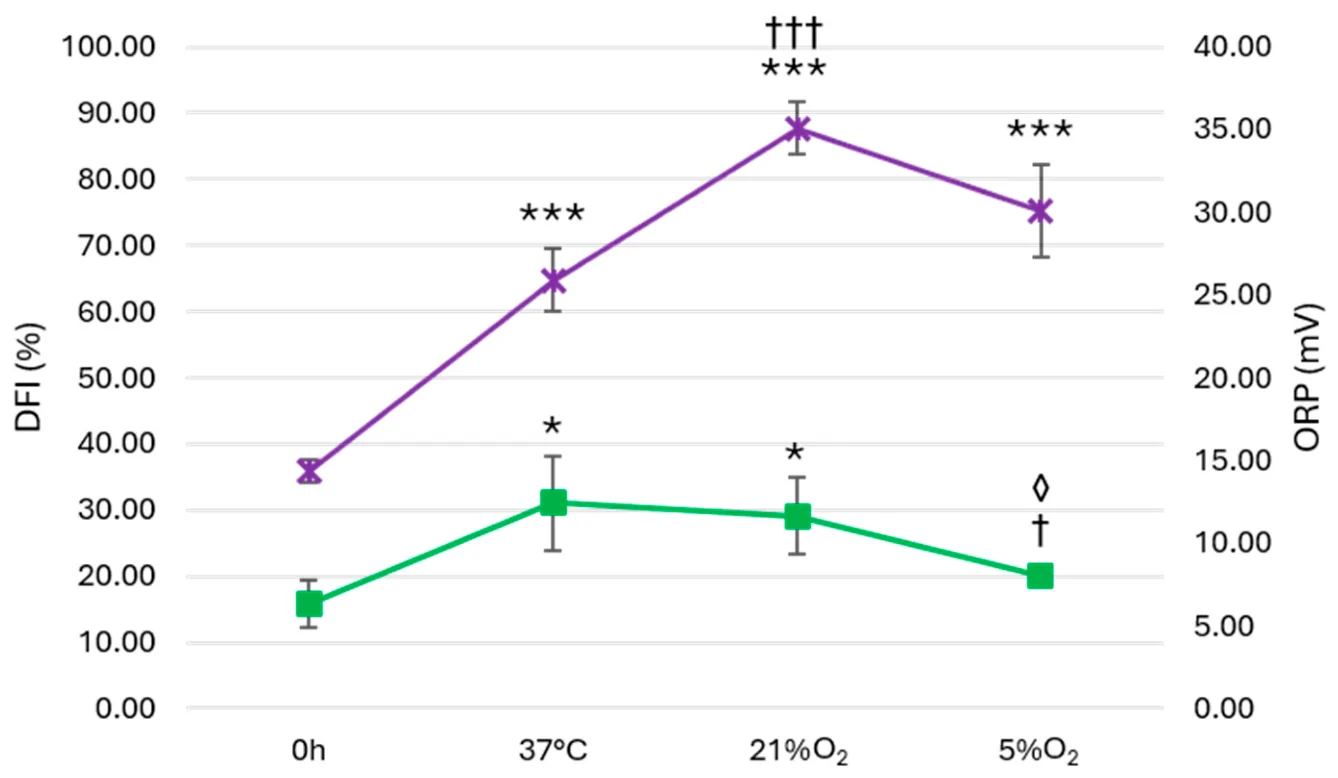
Effect of oxygen concentration (21 and 5%) on functional sperm parameters (ORP (mV) labeled with purple, DFI (%) with green; “asterisk” vs. 0 h, “dagger” vs. 37 °C, “diamond” vs. 21%O_2_ (Friedman-test, Bonferroni-Holm post hoc test, single “asterisk/dagger/diamond”: *p* < 0.05, triple “asterisk/dagger” *p* < 0.001).

Only exposure to 21% oxygen resulted in significantly higher DF values compared with the 0 h control (*p* < 0.05). When compared with 37 °C, only 5% O_2_ maintained DF at a significantly lower level (*p* < 0.05), whereas 21% O_2_ caused no significant difference. Similarly, 5% oxygen preserved DNA integrity significantly better than 21% oxygen (*p* < 0.05) (Figure 4).

### 2.3. Light Exposure

Both incubation in a dark environment (24 °C) and exposure to visible light significantly decreased total and progressive motility compared with the 0 h control, while UV exposure caused a drastic reduction in both parameters (*p* < 0.001). However, when the effects of the dark environment and visible light were compared, no significant differences were detected in either parameter. In contrast, UV treatment significantly decreased both total and progressive motility compared with either dark incubation or visible light exposure (*p* < 0.001).

**Figure 5:**
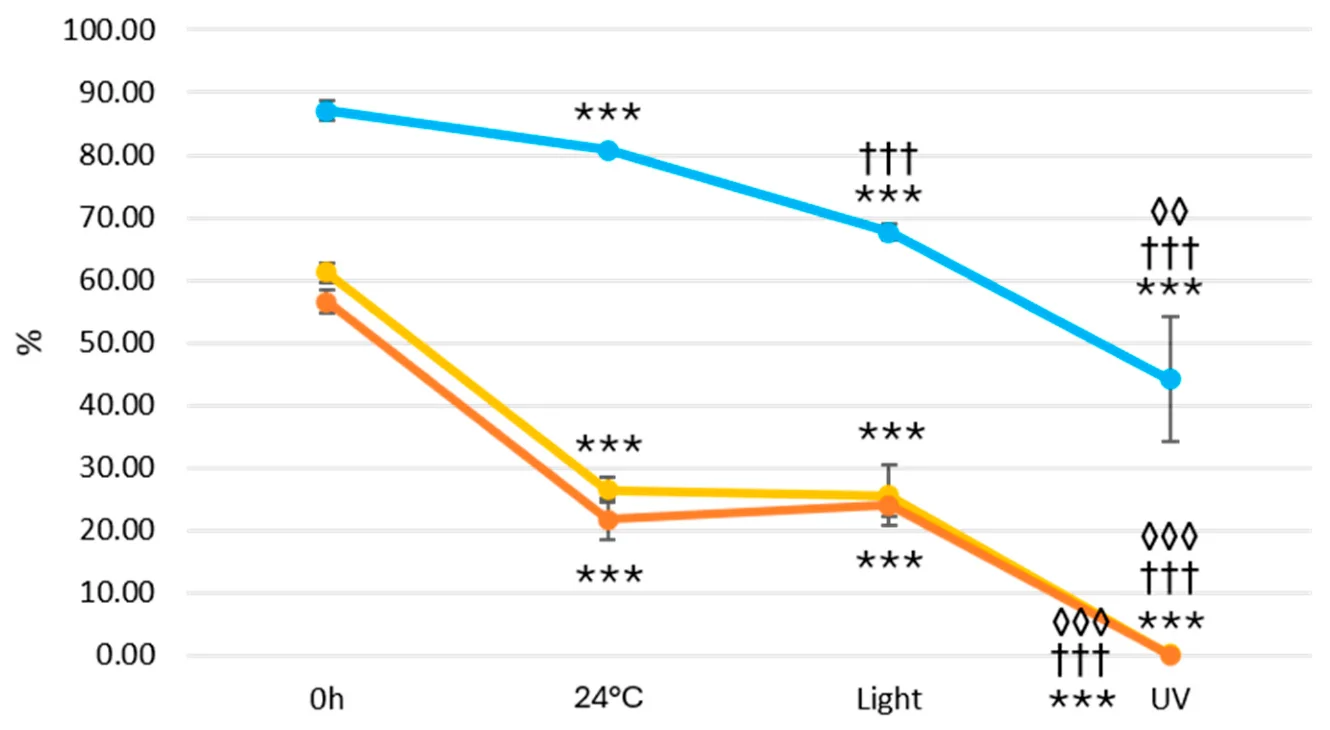
Effect of light exposure (visible light and UV) on classical sperm parameters (motility (%) labeled with yellow, progressive motility (%) with orange, vitality (%) with blue; “asterisk” vs. 0 h, “dagger” vs. 24 °C, “diamond” vs. light (Friedman-test, Bonferroni-Holm post hoc test, double “diamond”: *p* < 0.01, triple “asterisk/dagger/diamond”: *p* < 0.001).

Regarding vitality, all three lighting conditions (darkness, visible light, and UV) induced a statistically significant decline compared with the baseline (*p* < 0.001). When the effects of visible light and UV radiation were compared with those observed in the dark environment, significantly lower vitality values were detected after exposure to both light conditions (*p* < 0.001). If we compared the effect of UV to visible light, we could detect a further decline in vitality (*p* < 0.01) (Figure 5).

ORP showed a mild decrease after incubation in visible light and a pronounced increase after UV exposure compared with the 0 h control. Both changes were statistically significant (*p* < 0.05 and *p* < 0.001, respectively). Furthermore, ORP values after UV radiation were significantly higher than those measured in both 24 °C control and visible light (*p* < 0.001).

**Figure 6:**
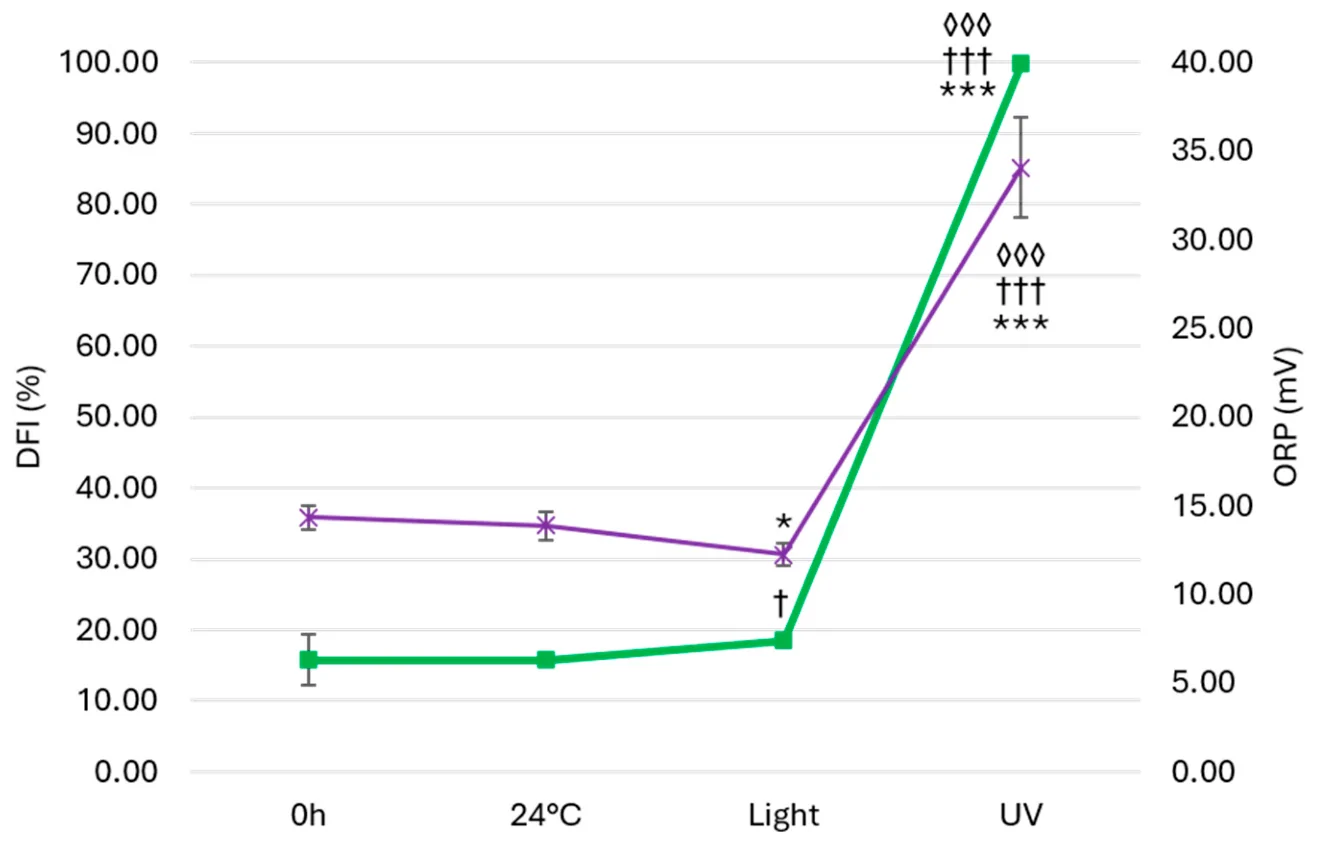
Effect of light exposure (visible light and UV) on functional sperm parameters (ORP (mV) labeled with purple, DFI (%) with green; “asterisk” vs. 0 h, “dagger” vs. 24 °C, “diamond” vs. light (Friedman-test, Bonferroni-Holm post hoc test, single “asterisk/dagger”: *p* < 0.05, triple “asterisk/dagger/diamond”: *p* < 0.001).

Only the UV-irradiated samples showed a significant elevation of DF compared with the 0 h control. Relative to the 24 °C control, incubation for 1 h in visible light already resulted in a significant increase (*p* < 0.05), whereas the UV-induced rise in DF was markedly higher (*p* < 0.001). UV irradiation caused further substantial elevation of DF compared to visible light (*p* < 0.001) (Figure 6).

### 2.4. Centrifugation

Exposure of the samples to centrifugation at 400× *g* for 1 h resulted in significantly lowered total and progressive motility compared with the 0 h control (*p* < 0.001). However, when compared with 24 °C, only a mild but significant decrease was observed in total motility (*p* < 0.05).

**Figure 7:**
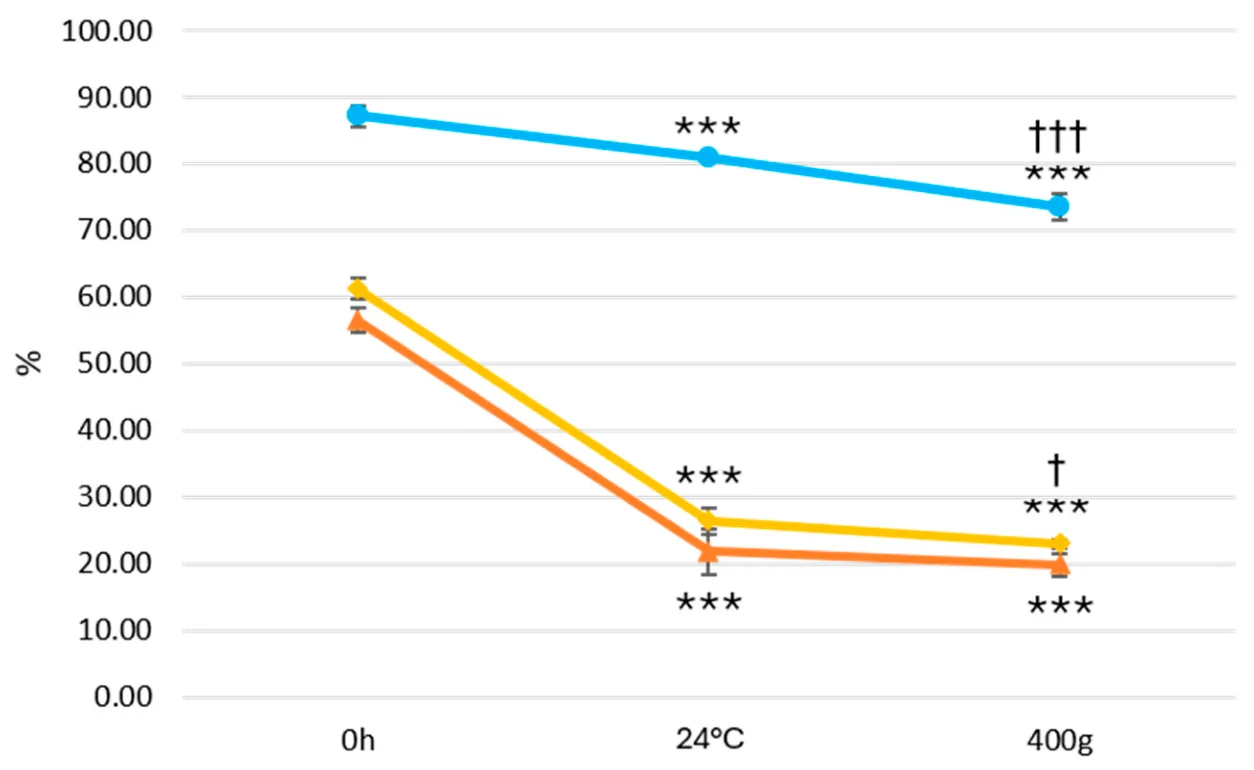
Effect of centrifugation (400× *g*) on classical sperm parameters (motility (%) labelled with yellow, progressive motility (%) with orange, vitality (%) with blue; “asterisk” vs. 0 h, “dagger” vs. 24 °C (Friedman-test, Bonferroni-Holm post hoc test, single “dagger”: *p* < 0.05, triple “asterisk/dagger”: *p* < 0.001).

Centrifugation significantly reduced vitality compared with both controls (0 h and 24 °C; *p* < 0.001) (Figure 7).

ORP did not change significantly after centrifugation relative to the aforementioned controls.

**Figure 8:**
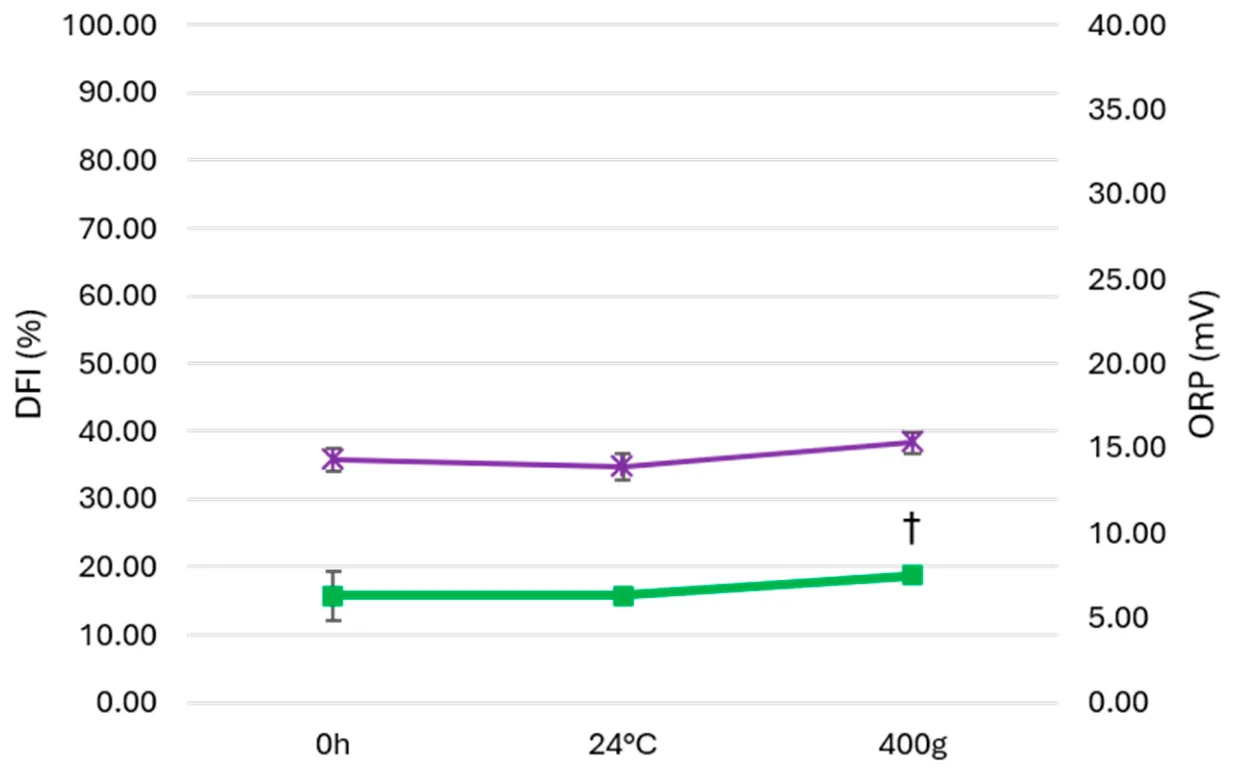
Effect of centrifugation (400× *g*) on functional sperm parameters (ORP (mV) labelled with purple, DFI (%) with green; “dagger” vs. 24 °C (Friedman-test, Bonferroni-Holm post hoc test, single “dagger”: *p* < 0.05).

DF increased slightly after centrifugation, reaching statistical significance only when compared with the second control (24 °C; *p* < 0.05) (Figure 8).

### 2.5. Relationship of ORP and DFI

**Figure 9:**
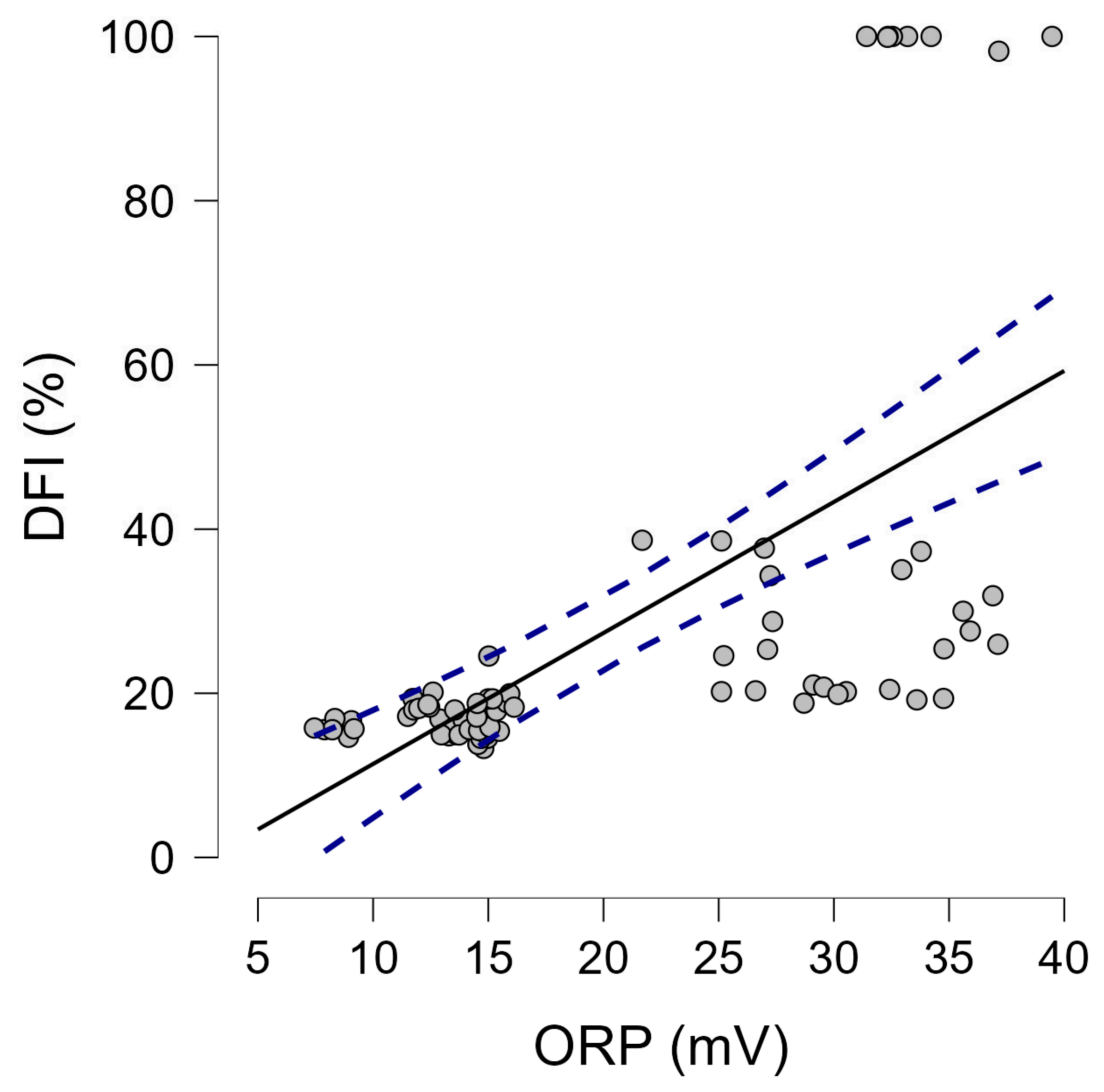
Scatter plot representing the correlation between ORP and DFI (Spearman correlation analysis, ρ = 0.732, *p* < 0.001). The solid grey line represents the fitted regression line, and the dashed navy lines represent the 95% confidence interval.

A Spearman correlation analysis was performed to reveal any correlation between ORP and DFI values. We discovered a strong positive correlation between the two variables (Spearman’s rho: 0.7316, *p* < 0.001) (Figure 9).

## 3. Discussion

As previously described, several external and internal factors can exert detrimental effects on classical sperm parameters, DNA integrity, and redox equilibrium, ultimately compromising overall sperm quality. These alterations, in turn, have significant implications for reproductive outcomes, as they influence multiple stages of the reproductive process, including fertilization, embryonic development, implantation, and clinical pregnancy. Villani et al. in 2021 performed a retrospective evaluation of 22.013 fresh ART cycles, highlighting sperm motility and morphology prediction of pregnancy and live birth rate (LBR) [23]. The same research group published a study about the clinical predictors of ART success: they compared 11.543 with fresh and 3.338 with frozen embryo transfers of 14.881 ART cycles. In case of fresh cycles, pregnancy rate (clinical and biochemical) and LBR were related to several female factors (age, number of injected/inseminated oocytes, and total embryos) and to progressive motility on the male side. In frozen cycles biochemical pregnancy rate remained related only to total embryos, while surprisingly, the clinical pregnancy rate and LBR showed correlation only with progressive motility in the case of IVF cycles [24]. Ribas-Maynou et al. published a systematic review (25.639 cycles) and meta-analysis (12.380 cycles) about the role of DF on ART outcomes. In IVF, higher DF showed correlation with lower fertilization, implantation, and pregnancy rates, embryo quality, and even LBR. These effects could not be extrapolated to intracytoplasmic sperm injection (ICSI) treatment: one possible explanation is that the embryologist chooses a single sperm for injection with good morphology and motility, which are known to be related to lower DF [25,26]. A recent umbrella meta-analysis by Wan et al., which included eight meta-analyses comprising more than 25.000 ART cycles, concluded that elevated DF was significantly associated with reduced clinical pregnancy rates following IVF and intrauterine insemination (IUI), whereas only a weak association was observed in ICSI. In contrast, elevated DF was strongly associated with pregnancy loss after ICSI. The available evidence regarding LBR remains limited and inconclusive [27]. Yang et al. demonstrated the influence of DF on embryonic development, revealing a lower blastocyst formation rate, a lower rate of transferable embryos, and a higher risk of low birthweight infants in the higher DF group [28]. Examining the reproductive outcomes of 144 patients with ICSI, Henkel et al. detected negative correlations between seminal ORP value and fertilization, blastocyst formation, implantation/clinical pregnancy, and LBR [29]. In IVF, Tomita et al. in 2023 showed a non-significant negative correlation between ORP and endpoints of IVF (clinical pregnancy rate, LBR) and a positive correlation with abortion rate [30]. Taken together, these findings highlight that sperm quality parameters, particularly motility, DNA fragmentation, and oxidative status, are associated with multiple clinically relevant reproductive outcomes, underscoring the importance of preserving sperm functional and genomic integrity during laboratory handling.

Sperm samples collected for diagnostic, therapeutic, or research purposes are exposed to various noxae, including fluctuations in temperature, light, gas composition (O_2_, CO_2_), pH, osmolarity, and mechanical forces such as centrifugation. According to the 6th edition of the WHO laboratory manual [31], spermatozoa should be separated from seminal plasma within 1 h of ejaculation to limit potential damage caused by non-sperm cells, such as increased exposure to ROS generated by leukocytes [32], as well as rising osmolarity during in vitro storage [33]. Earlier studies by Björndahl and Kvist (1990) demonstrated that the seminal vesicular fraction of seminal plasma may decrease zinc-dependent chromatin stability [34,35]. In addition, decapacitation factors present in seminal plasma inhibit the acquisition of hypermotility, which is essential for successful fertilization [36]. However, the role of seminal plasma is complex, as substantial evidence also supports its protective antioxidant function against oxidative stress [37,38]. Notably, our research group demonstrated in 2023 that exposure to multiple ROS-inducing agents resulted in more pronounced damage in seminal plasma-depleted samples, and highlighted the clinical relevance of ORP measurement using the MiOXSYS system [39].

Current international guidelines provide several recommendations for semen handling and laboratory environmental control. The WHO manual [31] recommends initiating semen analysis within 30–60 min after ejaculation and maintaining the sample at 37 °C until liquefaction is complete. The use of an orbital mixing platform is advised, although manual swirling is also acceptable. For microscopic examination, the use of a prewarmed slide and coverslip is recommended. For sperm preparation, the manual recommends the use of appropriate culture media, typically balanced salt solutions supplemented with protein (e.g., albumin) and appropriate buffering systems, particularly when the incubator contains atmospheric air and is maintained at 37 °C. During semen washing to remove seminal plasma, centrifugation at 500–600× *g* for 8–10 min may be used to achieve complete sperm pelleting. For specific sperm preparation techniques, different temperature recommendations are provided: 37 °C for swim-up and room temperature for magnetic-activated cell sorting, whereas no specific temperature is recommended for density-gradient centrifugation. For thawing cryopreserved sperm samples, the manual recommends 37 °C following conventional cryopreservation and 42–43 °C following vitrification. The ESHRE “Good practice in IVF laboratories” guideline [40] recommends avoiding extreme temperatures (<20 or >37 °C), initiating sperm preparation preferably within 30 min of semen collection, and regularly monitoring the temperature of incubators and heated platforms, as even deviations of ±1 °C may have a considerable impact on sperm motility parameters. The ASRM [41] guideline provides some general recommendations regarding laboratory environmental monitoring, lighting, equipment temperature control, and gas conditions for specific ART procedures. Taken together, these recommendations indicate that although current guidelines provide guidance on several aspects of sperm handling, specific quantitative recommendations regarding temperature, light exposure, and oxygen concentration for maintaining sperm quality during laboratory handling remain limited. Our findings, therefore, provide complementary experimental evidence on how routine laboratory conditions (24, 37 °C, visible light, 21%O_2_) or experimental stress conditions (4, −80 °C, 5%O_2_, UV, prolonged centrifugation (400× *g* for 1 h)) may affect sperm motility, vitality, oxidative status (ORP), and DF during laboratory handling. A summary of the effects of the investigated environmental conditions on the examined sperm parameters is provided in Table 1 and Table 2.

Semen samples are commonly handled at room temperature (24 °C) or at 37 °C when an incubator or temperature-controlled platform is used during preparation and may be exposed to visible light and atmospheric oxygen (~21% O_2_). In conventional CO_2_-in-air incubators, sperm cells may also be exposed to an atmosphere containing approximately 5% CO_2_ in air, corresponding to approximately 21% O_2_. In contrast, reduced-oxygen (tri-gas) incubators are increasingly used in ART laboratories, particularly for IVF and embryo culture, providing lower oxygen concentrations (~5% O_2_). The 4 °C condition was included as an experimentally relevant low-temperature stress condition. It reflects potential exposure of semen samples to inappropriate or uncontrolled cold temperatures during laboratory handling or transport. Although current recommendations advise avoiding temperatures below 20 °C, the effects of short-term exposure to such low temperatures on sperm quality remain insufficiently characterized. There is evidence that cooling can reduce metabolic activity and ROS production in spermatozoa, potentially slowing the depletion of cellular energy reserves and limiting oxidative damage [42]. Moreover, in veterinary reproductive medicine, short-term chilling of spermatozoa is routinely used before insemination, particularly in species or situations where spermatozoa are highly susceptible to cryopreservation-related damage [43]. Although 4 °C is not recommended as a routine storage temperature for human semen during semen analysis or ART procedures, these observations provided a biological rationale for including 4 °C as an experimental cooling condition in the present study. Exposure to −80 °C without a cryoprotective agent was included as an extreme cold-stress condition to assess the effects of severe temperature-induced cellular damage. Dark incubation was used as a reference condition because spermatozoa are not exposed to light during their physiological passage through the reproductive tract, whereas laboratory handling may involve exposure to visible light. UV irradiation was included as a positive control because of its well-established ability to induce oxidative stress, impair sperm function, and increase sperm DNA fragmentation. Prolonged centrifugation at 400× *g* for 1 h was included as an additional experimental stress condition to assess the effects of extended mechanical stress on sperm quality.

Temperature alterations exert complex, and sometimes paradoxical, effects on semen parameters. We used 24 °C as a second control in addition to the initial 0 h sample, since semen samples are generally maintained at room temperature under laboratory conditions. Storage at this temperature resulted in decreased motility, progressive motility, and vitality, without detectable changes in DF or ORP. Although 37 °C corresponds to physiological body temperature, it surprisingly deteriorated all examined parameters compared with the 0 h sample, as well as resulting in lower motility and increased DF and ORP even compared with samples stored at 24 °C. Heat stress induced by elevated temperature is known to impair semen parameters through several mechanisms. Increased metabolic activity may deplete energy substrates and promote the accumulation of harmful byproducts. It may also enhance mitochondrial ROS generation and reduce antioxidant activity, including SOD and GSH. These changes can promote lipid peroxidation, DNA fragmentation, and apoptosis. However, most of these findings have primarily been demonstrated in animal models [44,45,46,47,48]. In most mammals, the testes are located outside the body cavity and have a distinct thermoregulation mechanism resulting in a testicular temperature approximately 2–4 °C lower than core body temperature [49,50]. This lower temperature provides optimal conditions for normal spermatogenesis and sperm maturation. Storage at −80 °C resulted in a marked deterioration of sperm motility, progressive motility, and vitality compared with both the 0 h baseline and the 24 °C control group. This finding is consistent with the well-established effects of cryopreservation, during which intracellular ice crystals may form and damage cellular structures, including the sperm membrane. To minimize such damage, cryoprotective agents are typically added to reduce intracellular water content and thereby limit ice crystal formation. In the absence of adequate cryoprotection, cryoinjuries may occur, leading to decreased motility and viability, impaired DNA integrity, alterations in plasma membrane fluidity, and increased production of ROS [51,52]. In contrast, incubating at 4 °C improved motility and ORP compared to the baseline 0 h sample. Moreover, in comparison to 24 °C, we could detect elevated total and progressive motility, vitality, and ORP values. These findings suggest that storage at 4 °C may better preserve several sperm quality parameters than room-temperature incubation on the short term. Storage at low temperatures above the freezing point may be beneficial because it reduces sperm metabolic activity, thereby conserving energy reserves. Conflicting data can be found in the literature: Iranpour et al. compared the effects of 4–6 °C and 25 °C on human sperm: there was no detectable difference in motility, viability and DNA integrity between storage at 4–6 °C and 25 °C in the first two days of storage, however, storage longer than two days up to 12 days at 4–6 °C provided better results than storage at 25 °C [53]. In another study, Keshtgar and her colleagues found all changes in sperm motility and viability were the same under room temperature (22–27 °C) and cooling (4 °C) at 24 h, except the straight-line velocity, which was greater in the cooling group, but after 48 h, total motility and viability were lower in the cooled group. Therefore, cold preservation is recommended for 24 h and preservation at 22–27 °C is preferred for longer time storage [54]. Varner et al. [55] demonstrated that slow cooling (0.3 °C/min) followed by storage at 4 °C better preserved total and progressive motility in equine sperm than moderate or rapid cooling followed by storage at 4 °C. It also resulted in better motility than slow cooling followed by storage at 25 °C or storage at 37 °C. This indicates that the so-called „cold shock” caused by rapid cooling should be avoided. In an experimental study comparing incubation at 37 °C with 25 °C and 34 °C (representing physiological testicular temperature), significantly higher levels of phosphatidylserine externalization—an established marker of early apoptosis—and increased DF were observed following incubation at 37 °C [56]. Appell et al. investigated sperm viability and motility in samples obtained from fertile men undergoing vasectomy and maintained at 4, 20, or 37 °C for 3, 6, 12, and 18 h after collection. Both parameters deteriorated over time, with the decline being most pronounced at 37 °C and 20 °C. At 4 °C, motility progressively declined and was virtually absent after 18 h, whereas viability was relatively better preserved than at the higher temperatures [57].

Findings of this study showed that atmospheric oxygen concentration (21%) caused a significant reduction in sperm vitality and a concomitant increase in ORP, indicating enhanced oxidative stress. In contrast, incubation under 5% oxygen preserved sperm quality more effectively, resulting in improved total and progressive motility and lower DF, although vitality remained reduced compared with the 37 °C baseline sample. Direct comparison of the two oxygen concentrations further revealed significantly higher motility and vitality, together with lower DF values, under 5% oxygen than atmospheric oxygen. These findings suggest that physiological oxygen tension (5%) provides a more favorable environment for maintaining sperm function and genomic integrity during short-term laboratory handling. Griveau et al. could detect among oligozoospermic (but not normozoospermic) samples a positive effect of 5% oxygen tension above 21% as improved motility, survival rate, induced acrosome reaction and penetration of zona-free hamster eggs after capacitation [58]. To the best of our knowledge, there are no other existing human or animal studies directly evaluating the effects of in vitro oxygen tension on sperm quality under controlled laboratory conditions. Atmospheric oxygen concentration substantially exceeds the physiological oxygen tension encountered within the female reproductive tract, where oxygen levels generally range between 2% and 8% [59]. Consequently, prolonged exposure to ambient oxygen during laboratory procedures may create a relatively hyperoxic environment that enhances oxidative injury of sperm.

Our results demonstrate a profound reduction in motility, progressive motility, vitality, and elevation of ORP and DF values after UV exposure. These are consistent with the well-established effect of UV light to produce OS and direct DNA damage in sperm [60,61,62]. Torres et al. reported decreased motility and viability after UV-C exposure [63]. Rajput et al. demonstrated that short-term UV-A exposure reduces sperm viability, increases chromatin immaturity and toroid disruption, induces DF and lipid peroxidation, and lowers antioxidant enzyme activity, including SOD and CAT [5]. Amaral et al. [64] showed that UV-B irradiation leads to an increase in sperm mitochondrial ROS production and lipid peroxidation. Visible light appears to act through endogenous photosensitizers (e.g., cytochromes in mitochondria) to modulate ROS signaling, Ca^2+^ influx, and ATP production in sperm, thereby enhancing motility and fertilizing ability. Importantly, current evidence suggests that visible light does not generally impair sperm DNA integrity under the conditions studied [60,65]. Shahar et al. reported that visible light irradiation increased sperm hyperactivated motility through a mechanism involving ROS generation and epidermal growth factor receptor (EGFR) activation. In their mouse in vitro fertilization model, pre-irradiation of sperm also improved fertilization outcomes, with the number of fertilized oocytes increased by 74% [66]. Preece et al. demonstrated enhanced sperm motility without significant DNA damage after illumination with red laser light [67]. In contrast, we observed a reduction in sperm vitality and a modest increase in DF following exposure to visible light emitted by an ordinary ambient laboratory lamp. This discrepancy may be explained by differences in exposure duration. In the present study, sperm samples were exposed to visible light for 1 h, whereas Shahar et al. [66] employed only a 3-min exposure period. Prolonged illumination may increase the cumulative generation of reactive oxygen species through photosensitization reactions, resulting in detectable oxidative damage to sperm DNA and cellular structures. These effects have to be taken into consideration during the storage and optical micromanipulation of sperm in the IVF laboratory.

Relatively few studies have investigated the effects of centrifugation on human sperm function. Makler and Jakobi in 1981 were among the first to demonstrate that centrifugation at low centrifugal forces (<330× *g*) for 10–20 min did not significantly affect sperm motility, whereas higher centrifugal forces (>550× *g*) applied for the same duration resulted in a significant reduction in motility. [68]. Shekarriz et al. [69] evaluated the influence of centrifugation duration (2 vs. 10 min) and centrifugal force (200× *g* vs. 500× *g*) on ROS generation. They concluded that centrifugation time had a greater impact on ROS production than centrifugal force. The detrimental effect of increasing *g*-force became more evident during longer centrifugation periods. The authors proposed that ROS originated primarily from two sources: activated seminal leukocytes and a subpopulation of spermatozoa with compromised membrane integrity. Taken together, the currently available evidence suggests that centrifugation may negatively affect sperm quality, with centrifugation duration appearing to be a more critical determinant than centrifugal force.

An exploratory statistical analysis demonstrated a strong positive association between ORP and DFI, suggesting that increased oxidative stress is associated with increased sperm DNA damage under the investigated laboratory conditions. This finding is consistent with the established relationship between oxidative stress and sperm DNA damage described in the literature [70,71,72] and further supports the biological relevance of ORP as a marker of oxidative stress during laboratory sperm handling.

Several limitations should be considered when interpreting our findings. First, the relatively small sample size (*n* = 8) limits the generalizability of the results, although the repeated-measures design allowed each sample to serve as its own control and thereby reduced inter-individual variability. Second, the exposure period was limited to 1 h and therefore does not necessarily reflect longer laboratory handling or storage periods. Third, several investigated conditions, particularly −80 °C exposure and UV irradiation, represent experimental stress conditions rather than routine ART practice, and their effects should therefore not be directly extrapolated to clinical laboratory settings. Fourth, no clinical reproductive outcomes were assessed, and consequently, the observed changes in sperm parameters cannot be directly translated into fertilization, embryo development, or pregnancy outcomes. Furthermore, the visible-light and UV exposures were standardized with respect to the light source, exposure duration, and sample-to-source distance. However, the exact sample-level illuminance/irradiance and corresponding radiant exposure were not directly quantified. Finally, ORP normalization was not performed because the aim of the study was to assess the direct effect of environmental conditions on the measured oxidation-reduction potential within the same semen samples rather than compare oxidative status between different individuals. We acknowledge that normalized sORP values may provide additional information.

## 4. Materials and Methods

### 4.1. Semen Analysis According to the WHO Manual for the Laboratory Examination and Processing of Human Semen 5th Edition

#### 4.1.1. Classical Sperm Parameters

##### Motility

Liquefied semen samples were homogenized, and a native aliquot was examined under a coverslip by light microscopy at 400× total magnification. Spermatozoa were classified as progressive, non-progressive or immotile according to the 5th edition of the WHO Manual [20] for practical reasons. Motility was assessed by counting 200 spermatozoa across multiple fields.

##### Vitality

A drop of semen sample (5 μL) was mixed with an equal volume of 1% eosin-Y solution, a smear was made on a glass side, covered with a coverslip, incubated for 30 s at 37 °C, and 200 spermatozoa were evaluated. Unstained spermatozoa were classified as viable, while red-stained cells were considered non-viable. Vitality was expressed as the percentage of viable cells out of 200 counted.

##### Concentration

The first step was to select the appropriate dilution. A drop of the raw sample was placed on a slide and examined microscopically to determine the correct dilution ratio. The WHO manual provides guidance for choosing the appropriate initial and final volumes. To dilute the sample, we used the following fixative solution composition: 10 mL of 0.9% physiological saline, 25 µL of 3% hydrogen peroxide, and 50 µL of ortho-toluidine solution. After thorough mixing, 10 µL of the diluted sample was loaded into a Neubauer hemocytometer and left to settle for 10 min. Counting could then begin. At least 200 spermatozoa must be counted.

### 4.2. Determination of DNA Fragmentation

DF was assessed using the Sperm Chromatin Structure Assay (SCSA) with a commercially available kit (Cariad Medical Technology Co., Ltd., Zhuhai, China) according to the manufacturer’s instructions based on the original method described by Evenson et al. in 1999 [73]. The A, B, and C reagents were used as follows: the liquefied semen sample with known concentration was diluted to $1\times 10^{6}$ sperm/mL with Reagent A in a final volume of 100 µL in a 1.5 mL PCR tube. Subsequently, 200 µL of Reagent B was added, followed by gentle mixing for 30 s. Finally, 600 µL of Reagent C (prepared by mixing 1 mL of C1 with 6 µL of C2) was added, thoroughly mixed, and incubated in the dark for 5 min. Measurements were performed using a MACSQuant 16 flow cytometer (Miltenyi Biotec, Bergisch Gladbach, Germany) at 488 nm excitation. Spermatozoa with intact DNA emitted green fluorescence detected at 525 nm, while spermatozoa with fragmented DNA emitted red fluorescence detected at 680 nm. After gating out debris, the DFI was calculated as:

$$DFI=\frac{\mathrm{number}\;\mathrm{of}\;\mathrm{red}\text{-}\mathrm{fluorescent}\;\mathrm{cells}}{\mathrm{red}\;+\;\mathrm{green}\text{-}\mathrm{fluorescent}\;\mathrm{cells}}$$

### 4.3. Assessment of Oxidative Stress

Oxidative stress levels were inferred from the measurement of the ORP using the MiOXSYS (Caerus Biotechnologies, Geneva, Switzerland) system. This novel method employs a galvanostatic sensor that provides two values: at first, the so-called static ORP (sORP), which gives information about the initial ratio of oxidants and reductants in the semen. Afterwards, the device creates a low-voltage reducing current, inducing electron transfer from antioxidants to oxidants, yielding a value characterizing the antioxidant capacity reserve of the sample (cORP) [74].

In the current study, we used sORP values, which are referred to as ORP for simplicity. For measurement, 30 µL of the sample was applied to the sensor. ORP values were not normalized to sperm concentration because all parallel treatments used identical sperm concentrations. The measured millivolt values were compared across conditions.

### 4.4. Patient Selection, Experimental Setting

Semen specimens were collected by masturbation after at least 3 days of sexual abstinence from patients (*n* = 8) undergoing fertility evaluation. Normozoospermic samples (>15 M/mL) were included according to the WHO 5th edition reference limits [20]. This ensured exclusion of samples with pre-existing abnormalities that could bias responses to the treatments. Samples were collected with prior ethical approval and written informed consent from all participants.

After liquefaction and baseline assessment of semen parameters (concentration, vitality, motility, progressive motility, DF, ORP), each native semen sample was divided into ten tubes with dual-position caps (Vitrolife, Gothenburg, Sweden; 5 mL sample tube with two-position cap), each containing 200 µL aliquots. The special cap design set to open position allows gas exchange with the incubator atmosphere.

Applied treatments:

1. 0-h control (“0 h”): untreated, liquefied native ejaculate
2. 1-h room-temperature (24 °C) incubation, dark, closed cap (control, “24 °C”)
3. 1-h incubation at body temperature (37 °C), dark, closed cap (control for different oxygen concentrations, “37 °C”)
4. 1-h incubation at 4 °C, dark, closed cap (“4 °C”)
5. 1-h incubation at −80 °C, dark, closed cap (“−80 °C”)
6. 1-h incubation at 37 °C, dark, open cap, with atmospheric (21%) oxygen concentration (“21%O_2_”)
7. 1-h incubation at 37 °C, dark, open cap, with reduced (5%) oxygen concentration (“5%O_2_”)
8. 1-h room-temperature incubation under visible light (400–700 nm) emitted by an ordinary incandescent laboratory lamp (warm-white, 60 W, 2700 K, 806 lm), closed cap (“light”)

Exact illuminance, irradiance, and energy dose at the sample level were not quantified.

9. 1-h room-temperature incubation under UV-C light (Spectroline E-Series UV lamp (Supelco/Sigma-Aldrich, St. Louis, MO, USA; 6 W; 254 nm), 50 cm from the samples, open cap (“UV”)

The manufacturer does not provide sample-level irradiance for this model; therefore, the energy dose was not quantified. A typical peak intensity of 420 µW/cm^2^ at 15 cm has been reported for the corresponding 254 nm UV configuration but was not measured in our setup.

10. 1-h centrifugation at 400× *g*, dark, closed cap (“400× *g*”)

Following treatments, motility, progressive motility, vitality, DF, and ORP were reassessed. Sperm concentration was assumed constant throughout the experiment.

### 4.5. Statistical Analysis

Data distribution was assessed for each variable using the Shapiro–Wilk test, which indicated non-normal distribution in all cases. Accordingly, non-parametric statistical tests were applied. As each variable was measured under multiple environmental conditions, differences among treatment groups were evaluated using the Friedman test. When significant effects were detected, post-hoc pairwise comparisons were performed using the Bonferroni-Holm test. A two-tailed *p* value < 0.05 was considered statistically significant. No prior power analysis was performed because of the exploratory nature of the study. Nevertheless, to address the concern regarding sample size, we calculated effect sizes for the main outcomes using Kendall’s coefficient of concordance (W) for the Friedman tests. Very large effect sizes were observed for all primary outcomes (motility: W = 0.871, progressive motility: W = 0.913, vitality: W = 0.978, ORP: W = 0.942, DF: W = 0.895), indicating strong treatment effects despite the limited sample size. Statistical analyses were conducted using the open-source software JASP (version 0.96.0; University of Amsterdam, Amsterdam, The Netherlands), which is based on R (version 4.5.2).

## 5. Conclusions

UV irradiation and exposure to −80 °C, although not representative of routine ART practice, exerted the most detrimental effects on conventional sperm parameters. While −80 °C did not significantly affect ORP or DFI, UV irradiation caused the most pronounced increases in both parameters. These findings further emphasize the vulnerability of spermatozoa to environmental stress during laboratory processing. In contrast, under the conditions investigated, reduced oxygen tension (5% O_2_) and short-term exposure to 4 °C were associated with better preservation of some sperm quality parameters compared with the routine laboratory conditions. Additionally, an exploratory analysis revealed a strong positive correlation between ORP and DFI, further supporting the association between oxidative stress and sperm DNA damage.

From a practical perspective, our findings support minimizing unnecessary temperature fluctuations, prolonged exposure to atmospheric oxygen levels, excessive light exposure, and prolonged centrifugation during sperm handling. Furthermore, they highlight the need for well-defined environmental and handling parameters to support the preservation of sperm quality during assisted reproduction. However, such specifications for most of these parameters remain limited in current clinical guidelines.

## Figures and Tables

**Table 1:** Summary of the effect of environmental factors (temperature, light, centrifugal force) on classical and functional sperm parameters (1 vs. 0 h, 2 vs. 24 °C, 3 vs. light; Friedman-test, Bonferroni-Holm post hoc test, ↓/↑: *p* < 0.05, ↓↓/↑↑: *p* < 0.01, ↓↓↓/↑↑↑: *p* < 0.001; red: negative, green: positive change regarding sperm quality).

	24 °C	37 °C		4 °C		−80 °C		Light		UV			400 g	
	1.	1.	2.	1.	2.	1.	2.	1.	2.	1.	2.	3.	1.	2.
Motility	↓↓↓	↓↓↓	↓	↑	↑↑↑	↓↓↓	↓↓↓	↓↓↓	-	↓↓↓	↓↓↓	↓↓↓	↓↓↓	↓
Pr. mot.	↓↓↓	↓↓↓	-	↓↓↓	↑↑	↓↓↓	↓↓↓	↓↓↓	-	↓↓↓	↓↓↓	↓↓↓	↓↓↓	-
Vitality	↓↓↓	↓↓	-	↓↓	↑	↓↓↓	↓↓↓	↓↓↓	↓↓↓	↓↓↓	↓↓↓	↓↓	↓↓↓	↓↓↓
ORP	-	↑↑↑	↑↑↑	↓↓↓	↓↓↓	-	-	↓	-	↑↑↑	↑↑↑	↑↑↑	-	-
DF	-	↑	↑	-	-	-	-	-	↑	↑↑↑	↑↑↑	↑↑↑	-	↑

**Table 2:** Summary of the effect of the change of oxygen concentration on classical and functional sperm parameters (1 vs. 0 h, 4 vs. 37 °C, 5 vs. 21%O_2_; Friedman-test, Bonferroni-Holm post hoc test: ↓/↑: *p* < 0.05, ↓↓/↑↑: *p* < 0.01, ↓↓↓/↑↑↑: *p* < 0.001; red: negative, green: positive change regarding sperm quality).

	37 °C	21%O_2_		5%O_2_		
	1.	1.	4.	1.	4.	5.
Motility	↓↓↓	↓↓↓	-	↓↓↓	↑	↑↑
Pr. mot.	↓↓↓	↓↓↓	-	↓↓↓	↑↑↑	-
Vitality	↓↓	↓↓↓	↓↓↓	↓↓↓	↓↓	↑↑↑
ORP	↑↑↑	↑↑↑	↑↑↑	↑↑↑	-	-
DF	↑	↑	-	-	↓	↓

## Data Availability

Data are contained within the article. The raw data supporting the conclusions of this article will be made available by the authors on request.

## Abbreviations

The following abbreviations are used in this manuscript:

ART	Assisted reproductive technology
ASRM	American Society for Reproductive Medicine
CAT	Catalase
cORP	Capacity oxido–reduction potential
DF	DNA fragmentation
DFI	DNA fragmentation index
EGFR	Epidermal growth factor receptor
ESHRE	European Society of Human Reproduction and Embryology
GPX	Glutathione peroxidase
GSH	Glutathione
4HNE	4-hydroxynonenal
ICSI	Intracytoplasmic sperm injection
IUI	Intrauterine insemination
IVF	In vitro fertilization
LBR	Live birth rate
MDA	malondialdehyde
MOSI	Male oxidative stress infertility
OGG1	8-oxoguanine DNA glycosylase 1
ORP	Oxido–reduction potential
OS	Oxidative stress
PUFAs	Polyunsaturated fatty acids
ROS	Reactive oxygen species
SCSA	Sperm Chromatin Structure Assay
SOD	Superoxide dismutase
sORP	Static oxido–reduction potential
